# Abnormal pancreatic enzymes and their prognostic role after acute paraquat poisoning

**DOI:** 10.1038/srep17299

**Published:** 2015-11-25

**Authors:** Yi Li, Meng Wang, Yanxia Gao, Wen Yang, Qun Xu, Michael Eddleston, Li Li, Xuezhong Yu

**Affiliations:** 1 Emergency Department, Peking Union Medical College Hospital, Beijing 100730, China; 2 Department of Science and Technology, Beijing Tiantan Hospital, Capital Medical University, Beijing 100050, China; 3 Emergency Department, The First Affiliated hospital of Zhengzhou University, Zhengzhou 450052, China; 4 Institute of Medical Information, Chinese Academy of Medical Sciences & Peking Union Medical College, Beijing 100020, China; 5 Department of Epidemiology and Biostatistics, Institute of Basic Medical Sciences Chinese Academy of Medical Sciences, School of Basic Medicine Peking Union Medical College, Beijing 100005, China; 6 Pharmacology, Toxicology and Therapeutics, University of Edinburgh, Edinburgh, UK

## Abstract

Ingestion of paraquat causes multi-organ failure. Prognosis is best estimated through measurement of blood paraquat concentrations but this facility is not available in most hospitals. We studied the prognostic significance of abnormal pancreatic enzymes for survival. Patients with acute paraquat poisoning were recruited. An extensive series of blood tests including serum amylase were serially checked. Patients were sorted according to their serum amylase activity (normal [<220 U/L], mildly elevated [220 to 660 U/L], elevated [>660 U/L]), and survival compared between groups. 177 patients were enrolled to the study, of whom 67 died and 110 survived. 122 (70.62%), 27 (15.25%) and 25 (14.13%) patients were in the normal, mildly elevated and elevated amylase activity groups, respectively. The case fatality in the elevated group was 100% compared to 17% in the normal group (P < 0.001). We found four independent factors for paraquat death prediction: amylase, PaCO_2_, leukocyte number, and neutrophil percentage. Models using pancreatic enzyme activity showed good prediction power. We have found that abnormal pancreatic enzymes are useful prognostic marker of death after acute paraquat poisoning. Including serum amylase activity into a prognostic model provides a good prognostication.

Although paraquat is a highly effective herbicide, it is lethal after ingestion with mortality being around 80% after confirmed exposure^1^,^2^. The main cause of death is multi-organ failure including acute respiratory, renal and hepatic failure and cardiac injury^2^,^3^,^4^,^5^. Although pancreatic injury has been reported after paraquat poisoning^6^, it is unclear how often and when pancreatic injury occurs and whether it has any prognostic value.

Single lab analyses, such as serum paraquat concentration^1^,^7^,^8^,^9^, arterial lactate^10^, uric acid^11^, lymphocyte and neutrophil count, and creatinine^12^ have been used in risk stratification, and serum paraquat concentration has been thought as the most reliable parameter for prognosis prediction^9^. But it is difficult to extend the serum paraquat concentration technology to district hospitals because the high cost of the assay. Another potential indicator is the ingestion volume, which is affected by the difficulty of calculating it accurately and vomiting post-ingestion^8^,^13^.

Acute physiology and chronic health evaluation 2(APACHE 2)^14^, and sequential organ failure assessment (SOFA)^15^ scores had been used for the evaluation of critical patients. Some authors have studied their power in paraquat poisoning, showing APACHE 2 and SOFA to be helpful for paraquat poisoning mortality prediction^16^,^17^,^18^,^19^. The Poisoning Severity Score (PSS) has been recommended for stratification of poisoned patients^20^. But those scores are not easy for calculating in emergency department, where a simple and precise method of prognosis is needed.

We therefore prospectively studied pancreatic injury and other routine investigations in acute paraquat poisoning patients admitted to the first affiliated hospital of Zhengzhou University, a tertiary hospital in China. If independent risk factors can be found for prediction of death prediction, new models for prognosis prediction could be set up and compared with traditional score systems.

## Methods

Patients admitted to the first affiliated hospital of Zhengzhou University from July 2013 to August 2014 were enrolled.

Inclusion criteria were: acute paraquat poisoning by ingestion within 72 hours; confirmed by the semiquantitative urine paraquat test with positive result as the concentration was over 0.2 ug/mL (normal range 0–0.2 ug/mL)^21^; and consent from the patient or family. Patients were only enrolled if all 3 criteria were met.

The urine concentration was determined as the reported^21^: a standard line using urine from the healthy volunteer and the paraquat herbicide sample were drawn. Then put Na_2_CO_3_ and NaHCO_3_ to the urine sample to make pH > 9. The urine was filtered with film, and put Na_2_S_2_O_4_, and determine the urine paraquat concentration with spectrometry in 396 am.Then an equal was set up: urine paraquat concentration (ug/ml) = 5.1014* OD value, and with R^2^ = 0.9956.Concentration of fresh urine from the patient was checked and calculated using the equal as above.

Exclusion criteria were: poisoned by routes other than ingestion; admitted more than 72 hours after ingestion; co-ingestion of other toxins; pregnant or lactating patients; cardiac arrest after poisoning; past medical history of pancreas, heart, liver, kidney, or central nervous system disease; or refused consent. Patients were excluded if any one of the above criteria occurred.

All the patients were treated according to guidelines published by the China Physician Association (2013 version)^22^. All the patients received a single gastric lavage with room-warmed water regardless of whether they had been previously lavaged during this exposure. Six grams of smectite powder was given every 4 hours for the first day after admission.

Methylprednisolone 15 mg/kg/d was given and reduced one week later by 40 mg every 3 days. Ten to 15 mg/kg/d cyclophosphamide was administered for one week. Hemoperfusion was performed within 1 hour of admission for four hours, and repeated once a day for at least three days. Hemofiltration was performed for acute renal failure.

Patients were put on oxygen only when the pulse saturation was below 70% (or arterial oxygen pressure below 50 mm Hg), and dyspnea discomfort and signs had occurred.

The basic data of the patients were recorded, including age, gender, ingestion volume and time, the time of hemoperfusion and vital signs at admission. If the ingestion volume was unknown, it was estimated by mouthfuls (one female mouthful 30 ml, one male mouthful 40 ml).

Blood was drawn on the first, third, seventh and ninth day after admission for the routine lab tests, which included: serum amylase and lipase; platelet, leukocyte, lymphocyte and neutrophil percentage, plateletcrit, mean platelet volume; PaCO_2_ and PaO_2_, arterial lactate; alanine transaminase (ALT), total bilirubin, lactate dehydrogenase, γ-glutamyl transpeptidase; blood urea nitrogen and creatinine; cystatin C; creatine kinase, creatine kinase-MB. Glasgow coma score (GCS), APACHE 2, SOFA and PSS score were recorded for each patient after admission.

Based on the admission serum amylase activity, which normal range is 0–220 U/L, patients were sorted into three groups: normal group, with amylase lower than 220 U/L; mild amylase elevation group, with amylase between 220 U/L and 660 U/L; amylase elevation group, with amylase over 660 U/L.

### Ethical statement

All experimental protocols of the study were approved by the First Affiliated Hospital of Zhengzhou University’s ethics committee on June 29, 2013 with the number of ZY20130629, and were performed strictly as approved. The methods were carried out in accordance with the approved guidelines. Written informed consent was obtained from all enrolled patients.

### Statistical analysis

Data were expressed as median (25 quantile, 75 quantile) or as a percentage. Each variable above was compared between the survival and the deceased. Lifetest was used to compare survival curves of different pancreas groups. In order to analyze the prediction of the death of paraquat poisoning, logistic regression model and receiving operating characteristic (ROC) curve^23^ analyses were performed. Cox regression analysis was performed in order to build the prognostic indicator (PI) system. All *p*-values were two tailed, with statistical significance defined as *p* < 0.05. All statistical analyses were performed with SAS 9.2 software.

## Result

**Figure 1:**
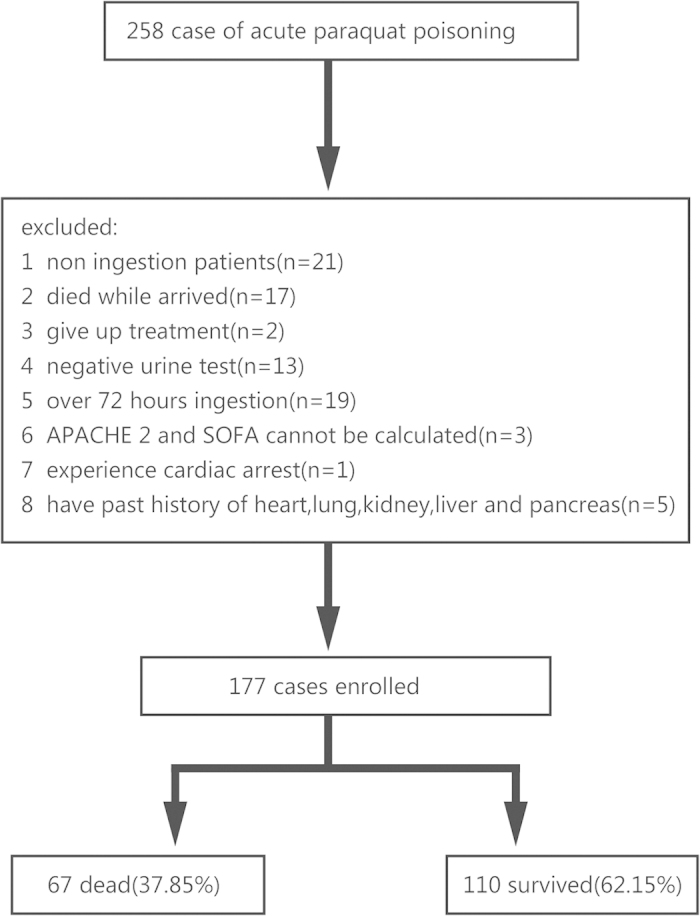
Flow chart of study recruitment.

Of 258 patients with acute paraquat poisoning admitted to our hospital, 177 had laboratory confirmation of paraquat ingestion within 72 hrs and were enrolled into the study (Fig. 1). There was a small male excess (97, 54.8% male vs 80, 45.2% female); their age ranged from one year to 66 years. The median ingestion volume was 30 (interquartile range 10 to 60) mL, with median urine paraquat concentration of 27.3 (interquartile range 5.1 to 72.9) ug/mL. Patients were admitted a median of seven (interquartile range 5 to 10) hours after ingestion (Table 1).

**Figure 2:**
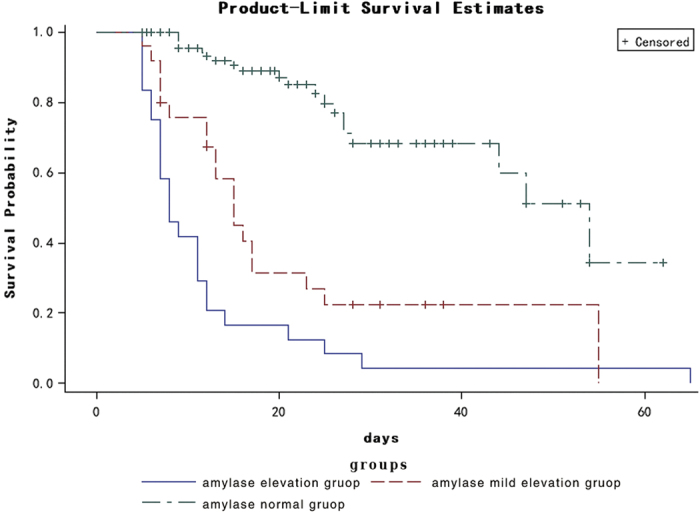
Survival curves for the groups according to the level of amylase. Blue solid line: elevated group; red dotted line: mildly elevated group; green dotted line: normal group.

Almost one third of patients had deranged pancreatic enzymes on admission. Twenty-seven patients had a mild elevation in amylase, while 25 had greater elevation (>660 U/L) and 125 had normal amylase. The case fatality in the elevation group was 100%, compared to 17% in the group without pancreatic enzyme changes (Table 2). Survival curves are depicted in Fig. 2, showing that deaths occurred later in the normal group, while deaths occurred most quickly in the elevation group, with significance difference among groups (P < 0.001).

**Figure 3:**
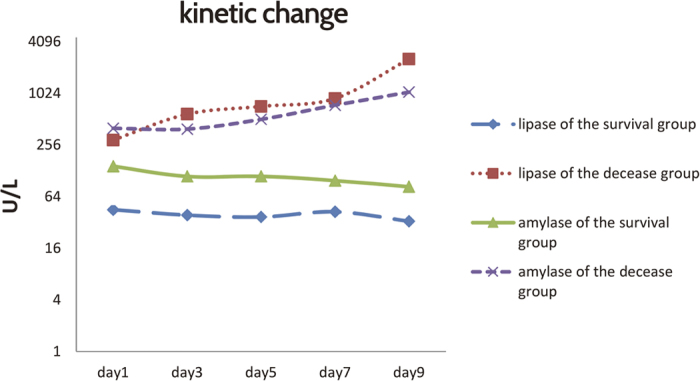
Daily kinetic change of amylase and lipase according to outcome. Red dotted line: lipase of the deceased group; purple dotted line: amylase of the deceased group; green solid line: amylase of the survival group; blue dotted line: lipase of the survival group. There were 48 patients in the survival group, and 18 patients in the deceased group.

An additional 14 patients developed pancreatic enzyme increase over the subsequent nine days, making a total of 66 patients (on admission, 27 had mild increase and 25 had greater increase; 14 increase in the following days.) with deranged pancreatic enzymes. These patients were sorted into a survival group (48 patients) and a deceased group (18 patients). The daily change of serum lipase and amylase is shown in Fig. 3. Both increased from admission until death; by contrast in the survival group they increased only a little and then remained at around this activity until discharge (P < 0.001 for the difference between groups). The kinetic changes of lipase were coincide with those of amylase, and there is positive relationship between the two pancreatic enzymes (r = 0.491, P < 0.0001).

No patient had abdominal pain. Imaging showed no evidence of pancreatic injury. Abdominal ultrasound was done on all patients, without finding of any abnormal results. A CT scan was done on patients with deranged pancreatic enzymes, but again no abnormality was noted. However, patients with deranged pancreatic enzymes developed abdominal distention, which was worse in those who died.

Univariate logistic regression analysis was done to select the predictors of death paraquat poisoning, and 10 predictors had P value lower than 0.05 were found (affiliated Table). Multivariate logistic regression analysis was performed to find out the independent index for the death, and leukocyte, amylase, neutrophil percentage and PaCO_2_ was chosen finally.

**Figure 4:**
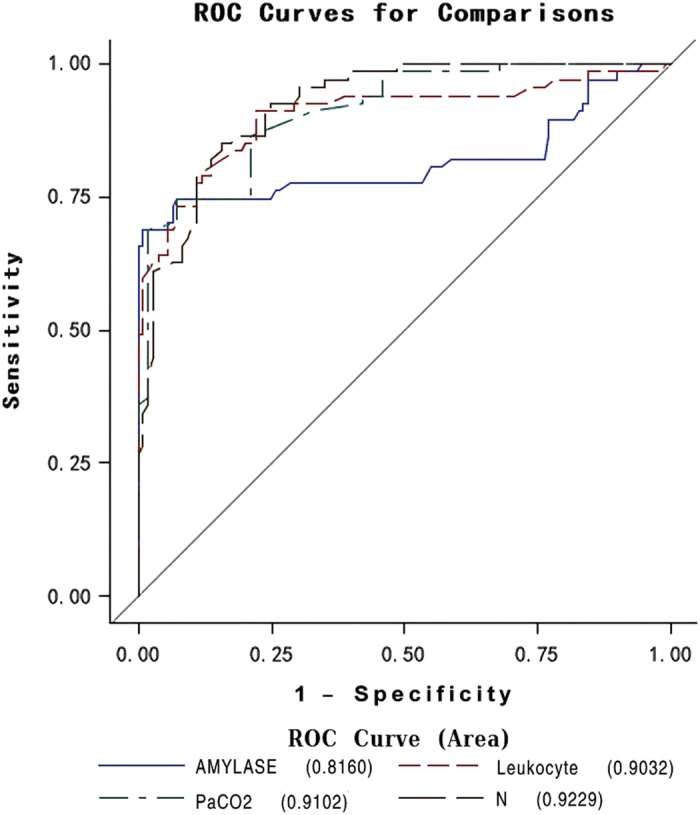
ROC for the factors with significant difference in the multi-logistic Regression.

ROC curve was drawn and area under the curve (AUC) were calculated for the four independent factors (Fig. 4). Neutrophil percentage had the highest AUC of 0.9229, while amylase had the lowest AUC of 0.8160 among the four factors.

We choose lipase and amylase in the equal of PI 1 and model was set up by Cox regression. PI 1: h(t) = h0(t)exp(0.0003571* lipase1 + 0.0004273* amylase1)

*PI1 of pancreatic enzymes* = (0.0003571* lipase1 + 0.0004273* amylase1).

Because the model above had not added other independent factors inside, we add neutrophil percentage, PaCO_2_, leukocyte count, and reserved amylase for pancreatic enzyme (excluding lipase). The second model, PI 2 based on the pancreas enzyme and other important factors, was made.

h(t) = h0(t)exp(0.08749 * N1 + 0.0004138* Amylase + 0.05096* WBC1-0.12222 * PaCO_2_).

*PI2* = (0.08749 * N1 + 0.0004138* Amylase + 0.05096* WBC1-0.12222 * PaCO_*2*_).

It was found that the PI 2 is the most precise one compared with the three traditional scores and PI 1, with AUC of 0.996 (Table 3, affiliated Figs 1 and 2).

## Discussion

In this study, we have focused on pancreatic injury in acute paraquat poisoning. The main findings of our study are: 1) pancreatic enzymes elevation occurs in paraquat poisoning; 2) the more severely deranged the pancreatic enzymes, the worse the outcome; 3) a prediction model utilizing amylase and leukocyte, neutrophil percentage and PaCO_2_, is much precise than other commonly used scores.

Acute paraquat poisoning can lead to multiple organ dysfunction syndrome (MODS), including lungs, gastrointestinal tract, kidney and liver, which commonly leads to death. Some previous studies had reported the abnormal pancreatic enzymes, but few studies have focused on the these changes or pancreas^6^,^10^,^24^,^25^,^26^,^27^,^28^,^29^. For example, a similar finding had been report that amylase together with PaCO_2_, and leukocyte has been found to be associated with the survival. But this paper had not focused on the clinical importance of amylase and had not set up a model based on these factors for survival prediction as us^30^.

The first evidence of pancreatic injury came from an autopsy report, which found an evident mild pancreatic change in one case^31^.Wang and colleagues reported a fatal case, in which serum amylase and lipase was increased several hours after ingestion and increased further thereafter. Afterwards, the lungs, kidney, liver and heart were involved till to the death^6^. So the authors thought that pancreas can be injured after acute paraquat poisoning and was related to the death.

A retrospective Chinese study of 502 cases admitted within 24 hours of paraquat ingestion reported that 180 (35.86%) cases with abnormal pancreatic enzymes, 171(95%) died, with only 9 (5%) being cured^28^. So we can see that there are high rate of abnormal pancreatic enzymes, which increase the death risk. But this report did not study the kinetic changes of pancreatic enzymes because it was a retrospective study.

Abnormal pancreatic enzymes have been observed in 10 cases in English literature^27^. In a study of retrospective 272 cases, Lee and colleagues showed that pancreatic enzymes were higher in patients who died than those who survived (median amylase 138.5 IU vs. 87 IU, lipase 37 IU vs 29 IU, respectively, both P < 0.0001)^10^. Another retrospective study of 296 cases found that elevated amylase activity was a significant predictor of survival using univariate analysis (deaths 480.5 ± 679.8 IU vs survivors 168.0 ± 181.9 IU, P < 0.01)^26^.

A single retrospective study has looked at pancreatic enzymes in 34 patients who survived paraquat poisoning^25^, which was the first study to focus on the abnormal pancreatic enzymes after paraquat ingestion. Pancreatic enzymes were elevated in 7 (20.6%) cases, and peaked on the seventh day; the extent of increase was positively related to the serum paraquat concentration on the fourth and seventh days (p < 0.05). CT examination was normal in all these patients, so the elevation was considered to be an inflammatory reaction by the authors^25^.

47 cases study in Chinese literature show that no patients had abnormal pancreatic enzymes after mild paraquat poisoning, while 58.5% had such increase after severe poisoning^32^. The increase rate in our study was 30%, which is higher than GIL HW’s report about alive paraquat patients alone (around 20%)^25^. The reason for the higher abnormal pancreatic enzymes rates in our study may be that ours are assumed more severe than theirs and is more close to the true clinical status.

Diagnostic criteria for acute pancreatitis include acute abdominal pain, a greater than 3 fold increase in pancreatic enzyme activity, and abnormal changes noted on ultrasound or CT scan. If two of the criteria were met, the diagnosis can be made^33^.

In view of one criteria of 3 fold increase amylase for acute pancreatitis diagnosis and the normal range of amylase is our hospital (0–220 U/L), we sorted the patients into three groups, i.e. normal (<220 U/L), 3 fold increases which satisfied the criteria for acute pancreatitis diagnosis (>660 U/L), increase but not satisfied for the diagnosis of 3 fold (>220 U/L, but <660 U/L). The best prognosis was found in normal group, and the worst in the elevation group. Because pancreatic enzymes elevation was found without abdominal pain and change on imaging, acute pancreatitis cannot be diagnosed.

The mechanism of the abnormal pancreatic enzymes changes is unclear. In the past, splanchnic hypoperfusion, drug adverse effects, stroke, cranial injury and others had been reported as the factors contributing to the changes^34^.The sources of amylase are the pancreas and salivary glands, intestine, and other tissues in small quantities, which may be identified by electrophoresis using isoform differentiation^35^. There are several lipases in the human body too, including lingual, pancreatic, lipoprotein, intestinal, and hepatic lipase. Between 11 and 12.5 percent of patients admitted to the hospital with non-pancreatic abdominal pain have an elevated serum lipase^36^. Because no abdominal pain and image changes can be found in our cases, so pancreatic injury or pancreatitis cannot be diagnosed at this stage. The abnormal pancreatic enzymes changes can be derived from the pancreas as well as the saliva, the intestine in paraquat poisoning, which need future exploration.

In view of the abnormal pancreatic enzymes changes in our study, we aimed to develop a prognosis model based around pancreatic enzymes. In the past, many experts had tried to find some single factors. Serum paraquat concentration is a good prognostic factor^37^,^38^.For example a recent report has retrospectively review 2136 paraquat ingestion patients, and set up three prediction models. The most powerful model of the three was composed of serum paraquat level and the ingestion time. But the limitations of the study were its retrospective design and the uneasy reach of serum paraquat test in clinical practice^39^.

Because the technology for performing the serum paraquat analysis is missing from most countryside hospitals, lactate^10^, uric acid^11^, lymphocyte and neutrophil count, and creatinine^12^ have been proposed as useful prognostic factors. But because single factors are easier to lead to errors, scores had been used, such as APACHE 2 and SOFA scores, and PSS^20^,^16^,^17^,^18^,^19^. But these three scores are time costing and need multiple laboratory results. Some easier scores, such as severity indexes related with serum paraquat concentration^40^ and the respiratory index (RI: A-aDO2/PO2 and the RI-time^41^ had been evaluated, but they have not been widely accepted. So the importance of finding easily applied practical scores requires attention.

In view of the unstable power of the single factors and the complex of the traditional scores, and different from most of the above retrospective study, our report is prospective design and our model is based on the most routine test in clinical work^39^.

We used multivariate logistic regression and identified four independent factors including leukocyte and neutrophil counts, PaCO_2_ and amylase for prediction of fatal paraquat poisoning. Prediction models were set up based on the pancreatic enzymes alone (PI 1) or on four independent factors (PI 2). The power of our prediction models was compared to traditional scores: PI 2 was found having the greatest power. In addition, the four lab exams in the PI 2 model are easy to do in nearly all the hospitals, so PI 2 model is recommended in future clinical work practice.

## Limitations

The serum paraquat cannot be checked and only urine paraquat level was checked in our study. The urine paraquat result may be influenced by the renal function^7^, so our PI 2 model needs the comparison of its power with serum and urine paraquat result in the future.

The cause of the abnormal pancreatic enzymes changes is unclear, and the pancreatic injury is needed to be confirmed in the future.

Larger sample are needed to validate our abnormal pancreatic enzymes changes and judge the power of our models.

## Conclusion

Acute paraquat poisoning can cause abnormal pancreatic enzymes changes which is often detectable soon after the ingestion and on admission to hospital. The more elevated the enzymes, the worse outcome of the patients. Amylase is an independent prognostic marker. Models including pancreatic enzymes have good prediction power, and are easier and simpler than the traditional scores.

## Additional Information

**How to cite this article**: Li, Y. *et al.* Abnormal pancreatic enzymes and their prognostic role after acute paraquat poisoning. *Sci. Rep.* **5**, 17299; doi: 10.1038/srep17299 (2015).

## Figures and Tables

**Table 1:** Patient demographics (n = 177).

Characteristic	value
Age (year, median IQR)	29 (22 to 43)
Male (%)	97 (56.1%)
GCS (median, IQR)	15 (14 to 15)
Ingestion volume (mL, median IQR)	30 (10 to 60)
Urine paraquat concentration (ug/mL, median IQR)	27.3 (5.1 to 72.9)
Delay from ingestion to admission (hr, median IQR)	7 (5 to 10)

IQR: interquartile range.

**Table 2:** Frequency of pancreatic injury.

Group	Number (%)	Deaths (% of all deaths)	Case fatality (95% CI)
Normal	125 (70.6)	21 (31.3)	0.17 (0.11 to 0.24)
Mildly elevated	27 (15.3)	21 (31.3)	0.78 (0.59 to 0.89)
Elevated	25 (14.1)	25 (37.3)	1.00 (0.87 to 1.00)

**Table 3:** Comparison of the prediction model with traditional scores.

	Best cutoff (>)	Sensitivity (%)	Specifity (%)	Diagnosis Accuracy (%)	Youden index	AUC
SOFA	9	77.61	87.27	83.62	0.65	0.889
PSS	3	100	98.18	98.87	0.98	0.990
APACHE2	14	97.01	93.64	94.92	0.91	0.975
PI1	0.46	77.61	99.08	90.1	0.767	0.903
PI2	0.18	100	96.33	99.6	0.963	0.996

PI1: model incorporating pancreatic enzymes including amylase and lipase.

PI2: model incorporating leukocyte, amylase, neutrophil percent (N%), and PaCO_2_.

## References

[b1] Gil H. W., Kang M. S., Yang J. O., Lee E. Y. & Hong S. Y. Association between plasma paraquat level and outcome of paraquat poisoning in 375 paraquat poisoning patients. Clin Toxicol (Phila). 46, 515–518 (2008). 18584363 10.1080/15563650701549403

[b2] Gawarammana I. B. & Buckley N. A. Medical management of paraquat ingestion. Br J Clin Pharmacol. 72, 745–57 (2011). 21615775 10.1111/j.1365-2125.2011.04026.x PMC3243009

[b3] Pavan M. Acute kidney injury following Paraquat poisoning in India. Iran J Kidney Dis. 7, 64–6 (2013). 23314145

[b4] Dinis-Oliveira R. J. *et al.* Paraquat poisonings: mechanisms of lung toxicity,clinical features, and treatment. Crit Rev Toxicol. 38, 13–71 (2008). 18161502 10.1080/10408440701669959

[b5] Bismuth C., Garnier R., Baud F. J., Muszynski J. & Keyes C. Paraquat poisoning. An overview of the current status. Drug Saf. 5, 243–51 (1990). 2198050 10.2165/00002018-199005040-00002

[b6] Wang L. & Qian Y. Y. A deceased case report of paraquat ingestion induced severe pancreatic injury. Zhong Guo Yao Wu Ying Yong He Jian Che 1, 37–38 (2005). (in Chinese)

[b7] Scherrmann J. M., Houze P., Bismuth C. & Bourdon R. Prognostic value of plasma and urine paraquat concentration. Hum Toxicol. 6, 91–3 (1987). 3817835 10.1177/096032718700600116

[b8] Hart T. B. & Nevitt A., Whitehead,A. A new statistical approach to the prognostic significance of plasma paraquat concentrations. Lancet. 2, 1222–1223 (1984). 6150271 10.1016/s0140-6736(84)92784-3

[b9] Senarathna L. *et al.* Prediction of outcome after paraquat poisoning by measurement of the plasma paraquat concentration. QJM. 102, 251–259 (2009). 19228776 10.1093/qjmed/hcp006 PMC2659600

[b10] Lee Y., *et al.* Arterial lactate as a predictor of mortality in emergency department patients with paraquat intoxication. Clin Toxicol (Phila). 50, 52–6 (2012). 22175790 10.3109/15563650.2011.639716

[b11] Zhang J. *et al.* The significance of serum uric acid level in humans with acute paraquat poisoning. Sci Rep. 5, 9168 (2015). 25772718 10.1038/srep09168 PMC4360628

[b12] Kang C. *et al.* Absolute lymphocyte count as a predictor of mortality in emergency department patients with paraquat poisoning. PLoS One. 8, e78160 (2013). 24205140 10.1371/journal.pone.0078160 PMC3813447

[b13] Wilks M. F. *et al.* Improvement in survival after paraquat ingestion following introduction of a new formulation in Sri Lanka. PLoS Med. 5, e49 (2008). 18303942 10.1371/journal.pmed.0050049 PMC2253611

[b14] Knaus W. A., Draper E. A., Wagner D. P. & Zimmerman J. E. APACHE II: A severity of disease classification system. J Crit Care Med. 13, 818–29 (1985). 3928249

[b15] Moreno R. *et al.* The use of maximum SOFA score to quantify organ dysfunction/failure in intensive care. Results of a prospective, multicentre study. Working Group on Sepsis related Problems of the ESICM. Intensive Care Med. 25, 686–96 (1999). 10470572 10.1007/s001340050931

[b16] Huang N. C., Lin S. L., Hung Y. M., Hung S. Y. & Chung H. M. Severity assessment in acute paraquat poisoning by analysis of APACHE II score. J Formos Med Assoc. 102, 782–787 (2003). 14724724

[b17] Huang N. C. *et al.* Further evidence of the usefulness of Acute Physiology and Chronic Health Evaluation II scoring system in acute paraquat poisoni*ng*. Clin Toxicol (Phila). 44, 99–102 (2006). 16615662 10.1080/15563650500514251

[b18] Weng C. H. *et al.* Sequential organ failure assessment score can predict mortality in patients with paraquat intoxication. PLoS One. 7, e51743 (2012). 23272154 10.1371/journal.pone.0051743 PMC3522704

[b19] Weng C. H. *et al.* Predictors of acute respiratory distress syndrome in patients with paraquat intoxication. PLoS One. 8, e82695 (2013). 24349340 10.1371/journal.pone.0082695 PMC3859634

[b20] Persson H., Sjöberg G., Haines J. & Pronczuk G. J. Poisoning Severity Score: Grading of acute poisoning. J Toxicology - Clinical Toxicology. 36, 205–13 (1998). 10.3109/15563659809028940 9656975

[b21] Shiyi Sun, Cui Wang & Xiaofang Luo Determination of paraquat in urine with spectrometry method. Chinese Journal of Health Laboratory Technology. 18, 819–821 (2008). (in Chinese)

[b22] China Doctor Association of Emergency. Consensus of acute paraquat diagnosis and treatment. China Journal of Critical Care Medicine. 33, 484–490 (2013). (in Chinese)

[b23] Youden W. J. Index for rating diagnosis tests. Cancer. 3, 32–35 (1950). 15405679 10.1002/1097-0142(1950)3:1<32::aid-cncr2820030106>3.0.co;2-3

[b24] Yang Q., Liu Y. H., Hu Q., Jiang W. W. & Zhang X.Z. A case report of ventricular arrithymia induced by acute severe paraquat poisoning. Jun Shi Yi Xue Yuan Yuan Kan. 34, 4 (2010). (in Chinese)

[b25] Gil H. W., Yang J. O., Lee E. Y. & Hong S. Y. The level and clinical significance of pancreatic enzymes in survivors of acute paraquat poisoning. Clin Toxicol (Phila). 47, 308–11 (2009). 19514877 10.1080/15563650902834497

[b26] Yang J. O., Gil H. W., Kang M. S., Lee E. Y. & Hong S. Y. Serum total antioxidant statuses of survivors and nonsurvivors after acute paraquat poisoning. Clin Toxicol (Phila). 47, 226–9 (2009). 18788002 10.1080/15563650802269901

[b27] Kang M. S., Gil H. W., Yang J. O., Lee E. Y. & Hong S. Y. Comparison between kidney and hemoperfusion for paraquat elimination. J Korean Med Sci. 24, S156–160 (2009). 19194546 10.3346/jkms.2009.24.S1.S156 PMC2633192

[b28] Cui W. H., Zhang X. R., Sun C.W. & Qiu Z.W. The complications analysis of 502 cases of acute paraquat poisoning. Zhong Guo Yi Kan. 48, 64–66 (2013). (in Chinese)

[b29] Zhang X. W. & Li Q. H. Serum change of amylase in acute paraquat poisoning. Qccupation and health. 23, 1074–75 (2007). (in Chinese)

[b30] Lee E. Y., Hwang K. Y., Yang J. O. & Hong S. Y. Predictors of survival after acute paraquat poisoning. Toxicol Ind Health. 18, 201–6 (2002). 12974543 10.1191/0748233702th141oa

[b31] Soontornniyomkij V. & Bunyaratvej S. Fatal paraquat poisoning: a light microscopic study in eight autopsy cases. J Med Assoc Thai. 75, S98–S105 (1992). 1402491

[b32] Zhang W. *et al.* Analysis of clinical classification and outcome of patients with acute paraquat poisoning. Chinese Journal of Emergency Medicine. 19, 357–360 (2010). (in Chinese)

[b33] Tenner S., Baillie J., DeWitt J. & Vege S.S. American College of Gastroenterology guideline: management of acute pancreatitis. Am J Gastroenterol. 108, 1400–15 (2013). 23896955 10.1038/ajg.2013.218

[b34] Chen C. C. Clinical implication of increased pancreatic enzymes in ICU patients. J Chin Med Assoc. 73, 129–30 (2010). 20230996 10.1016/S1726-4901(10)70026-5

[b35] Pieper B. C., Strocchi A. & Levitt M. D. Where does serum amylase come from and where does it go? Gastroenterol Clin North Am. 19, 793–810 (1990). 1702756

[b36] Gumaste V. V., Roditis N., Mehta D. & Dave P.B. Serum lipase levels in nonpancreatic abdominal pain versus acute pancreatitis. Am J Gastroenterol. 88, 2051–2055 (1993). 7504396

[b37] Min Y. G. *et al.* Prediction of prognosis in acute paraquat poisoning using severity scoring system in emergency department. Clin Toxicol (Phila). 49, 840–5 (2011). 22077247 10.3109/15563650.2011.619137

[b38] Jones A. L., Elton R. & Flanagan R. Multiple logistic regression analysis of plasma paraquat concentrations as a predictor of outcome in 375 cases of paraquat poisoning. QJM. 92, 573–8 (1999). 10627878 10.1093/qjmed/92.10.573

[b39] Hong S. Y., Lee J. S., Sun I. O., Lee K. Y. & Gil H. W. Prediction of Patient Survival in Cases of Acute Paraquat Poisoning. PLoS ONE. 9, e111674 (2014). 25415592 10.1371/journal.pone.0111674 PMC4240538

[b40] Suzuki K. *et al.* Evaluation of severity indexes of patients with paraquat poisoning. Hum Exp Toxicol. 10, 21–23 (1991). 1673620 10.1177/096032719101000104

[b41] Suzuki K. *et al.* A new method for predicting the outcome and survival period in paraquat poisoning. Hum Toxicol. 8, 33–38 (1989). 2714808 10.1177/096032718900800106

